# Predicting conversion to Alzheimer’s disease in individuals with Mild Cognitive Impairment using clinically transferable features

**DOI:** 10.1038/s41598-022-18805-5

**Published:** 2022-09-16

**Authors:** Ingrid Rye, Alexandra Vik, Marek Kocinski, Alexander S. Lundervold, Astri J. Lundervold

**Affiliations:** 1 Department of Biological and Medical Psychology, University of Bergen, Bergen, Norway; 2 Mohn Medical Imaging and Visualization Centre (MMIV), Department of Radiology, Haukeland University Hospital, Bergen, Norway; 3 Department of Biomedicine, University of Bergen, Bergen, Norway; 4 Institute of Electronics, Lodz University of Technology, Lodz, Poland; 5 Department of Computer Science, Electrical Engineering, and Mathematical Sciences, Western Norway University of Applied Sciences, Bergen, Norway

**Keywords:** Dementia, Alzheimer's disease, Human behaviour

## Abstract

Patients with Mild Cognitive Impairment (MCI) have an increased risk of Alzheimer’s disease (AD). Early identification of underlying neurodegenerative processes is essential to provide treatment before the disease is well established in the brain. Here we used longitudinal data from the ADNI database to investigate prediction of a trajectory towards AD in a group of patients defined as MCI at a baseline examination. One group remained stable over time (sMCI, n = 357) and one converted to AD (cAD, n = 321). By running two independent classification methods within a machine learning framework, with cognitive function, hippocampal volume and genetic APOE status as features, we obtained a cross-validation classification accuracy of about 70%. This level of accuracy was confirmed across different classification methods and validation procedures. Moreover, the sets of misclassified subjects had a large overlap between the two models. Impaired memory function was consistently found to be one of the core symptoms of MCI patients on a trajectory towards AD. The prediction above chance level shown in the present study should inspire further work to develop tools that can aid clinicians in making prognostic decisions.

## Introduction

Alzheimer’s disease (AD) is by far the most common type of dementia, estimated to account for 60–70% of all dementia cases^1^. The disease is characterized by an insidious onset caused by neurodegenerative processes, which lead to progressive loss of cognitive and functional abilities. Alongside the devastating personal consequences AD has on those affected and their caregivers, economical costs related to the disease are massive. In USA alone, the costs related to AD are estimated to reach $321 billion in 2022^2^. And this is just the tip of the iceberg. With the demographic composition of the population being skewed towards an increasing proportion of elderly, we are facing what has been described an AD-epidemic^3^, and related costs are predicted to more than triple within the year 2050^2^.

One of the difficulties for successful treatment of AD is the fact that its pathological hallmarks (i.e., amyloid beta and neurofibrillary tangles of tau proteins) tend to be established in the brain decades prior to the time a person’s cognitive and functional impairments are severe enough to get medical attention^4^. Management of known risk factors for AD (e.g., high blood pressure and diabetes) is therefore emphasized. Moreover, several recent studies point towards promising life-style interventions reducing AD-pathology and neurodegeneration and delaying symptom-onset (see e.g.,^5^). Taken together, much effort is put into early identification and treatment of patients in the prodromal phase of the disease. Mild Cognitive Impairment (MCI) has become a diagnostic concept to describe this phase^6^. Individuals falling within this diagnostic category show a cognitive decline greater than expected in normal cognitive aging, but still not with the severity of functional impairment characterizing those with dementia^7^.

Over the past two decades, several studies have shown that MCI comprises a heterogeneous patient group. This is true both with respects to clinical phenotypes and individual disease trajectories. Their clinical presentation is typically classified into an amnesic (aMCI) or non-amnesic type, and may affect a single or multiple cognitive domains^7^. According to the original description of MCI^8^, an impairment is defined when performance on a given psychometric test is at least 1.5 standard deviations below the expected mean for a given patient. An impairment in a patient may thus be defined as aMCI when results on a memory test is substantially lower than expected from sex and age corrected test norms or estimates of his/her general intellectual level. To support this definition, a clinical examination may also include brain measures of memory related structures like hippocampus, and sometimes also genetic analysis, where the presence of the APOE-e4 risk allele is known as the most reliable marker^9–11^.

It has been shown that patients with an aMCI diagnosis are more likely to progress to AD than patients in a non-aMCI subgroup^7,12^, with an annual conversion rate estimated to be 10–15%^9^. Others will remain stable over years, and some may even revert back to normal cognition in cases where somatic diseases or psychiatric disorders causing mild cognitive impairment are successfully treated^13^. Differentiating between cognitive changes characterizing incipient AD and a more stable or fluctuating pattern of cognitive impairment is therefore an important endeavour in the research field^14^.

Machine learning (ML) has in this context been established as an effective tool for making prognostic predictions in AD^15^, with several algorithms classifying stable MCI versus converting MCI subjects with impressive accuracy^16,17^. Despite this, translation into clinical practice has to a large degree been lacking. There are several factors contributing to this^18^, with one crucial obstacle being that most of these algorithms are constructed using data that are expensive and/or invasive to obtain. Although the inclusion of more invasive biomarkers^19,20^ and/or longitudinal data^21–23^ would increase the predictive power of the algorithms, this information is rarely obtained in an initial clinical examination of a MCI subject. To the best of our knowledge, few studies have aimed at creating classification models based on clinically relevant features, with a study from Grassi and colleagues^24^ being an exception. Their algorithm predicted conversion from MCI to AD with a balanced accuracy of 78% when sociodemographic and clinical characteristics were included as predictors. As indicated by the authors, scores from the neuropsychological tests used to define “ground truth” labels (i.e. stable or converters) were also included as predictors, which potentially could lead to inflated predictive performance due to circularity.

The short review presented above inspired the current study to further investigate predictive models of trajectories from MCI to AD. Longitudinal data were used to identify two groups of patients who were diagnosed with MCI at a baseline clinical examination: one group including patients who were diagnosed with AD and one group retaining their MCI diagnosis during the observation period. With an aim to make the results relevant to diagnostic decisions, we included features commonly used as part of an assessment of older adults presenting cognitive problems. These features included demographic data, information from neuropsychological and Magnetic Resonance Imaging (MRI) examinations and genetic information about APOE status. These features were used to train two different supervised learning algorithms to classify the patients into the two predefined groups: (1) an ensemble-based model constructed by combining five different models, and (2) a Random Forest (RF) model^25^. The results from the two models were compared, and the RF model used to identify feature importance^26^. The complex nature of MCI and AD surely leave us to expect misclassifications. We therefore explore clinical characteristics of the prediction labels (i.e true negative, false positive, false negative and true positive) returned from the most accurate model.

## Results

A total of 708 subjects defined as MCI at baseline met the inclusion criteria for the current study. From this, 30 subjects (24 sMCI, 6 cAD) had missing data points on at least one of the features used in model construction. These subjects were removed from further analyses, resulting in a final sample comprising 357 sMCI and 321 cAD subjects. Exploratory analysis showed that all features deviated from a normal distribution, and non-parametric analyses were therefore added. These analyses yielded similar results as the descriptive and comparative statistics presented in Table 1, with no differences in statistical significance. At baseline, the converters to AD showed significantly lower results on all included cognitive tests, lower hippocampus volumes and a higher number of APOE-4 carriers than the stable MCI group.

**Table 1:** Demographics, cognitive function and biological measures in patients defined as stable MCI and converters to AD.

	sMCI (N = 357)	cAD (N = 321)	t/$x^2$	p value	Effect size
	Mean (SD)	Mean (SD)			
**Demographics**					
Age	73.1 (7.45)	73.9 (7.11)	1.35	0.176	0.10
Gender (%F)	41.2	38.9	0.352	0.553	0.02
**Cognitive function**					
RAVLT-Im	36.9 (10.5)	29.3 (7.7)	10.56	< 0.001	0.81
RAVLT-delay	4.88 (3.93)	2.05 (2.67)	10.87	< 0.001	0.84
RAVLT-recog	11.26 (3.16)	9.42 (3.56)	7.13	< 0.001	0.55
TMTA	39.2 (15.6)	44.7 (21.5)	3.90	< 0.001	0.30
TMTB	108.1 (56.9)	133.8 (73.9)	5.10	< 0.001	0.39
CFT animals	17.8 (5.17)	15.8 (4.75)	5.13	< 0.001	0.39
GDS: mean (SD)	1.71 (1.44)	1.65 (1.38)	0.53	0.596	0.04
ANART Total errors	12.9 (9.3)	13.3 (9.6)	0.61	0.539	0.05
**Biological measures**					
Hippocampus volume	0.00451 (7.6$*10^{-4}$)	0.00398 (6.8$*10^{-4}$)	9.64	< 0.001	0.74
APOE (%positive)	42.3	64.2	32.45	< 0.001	0.22

*sMCI* stable Mild Cognitive Impairment, *cAD* converted to Alzheimer’s disease, *RAVLT* Rey Auditory Verbal Learning Test, *TMT* Trail Making Test, *CTF* Category Fluency Test, *GDS* Geriatric Depression Scale, *ANART* American National Reading Test.

### Performance of classification algorithms

**Figure 1:**
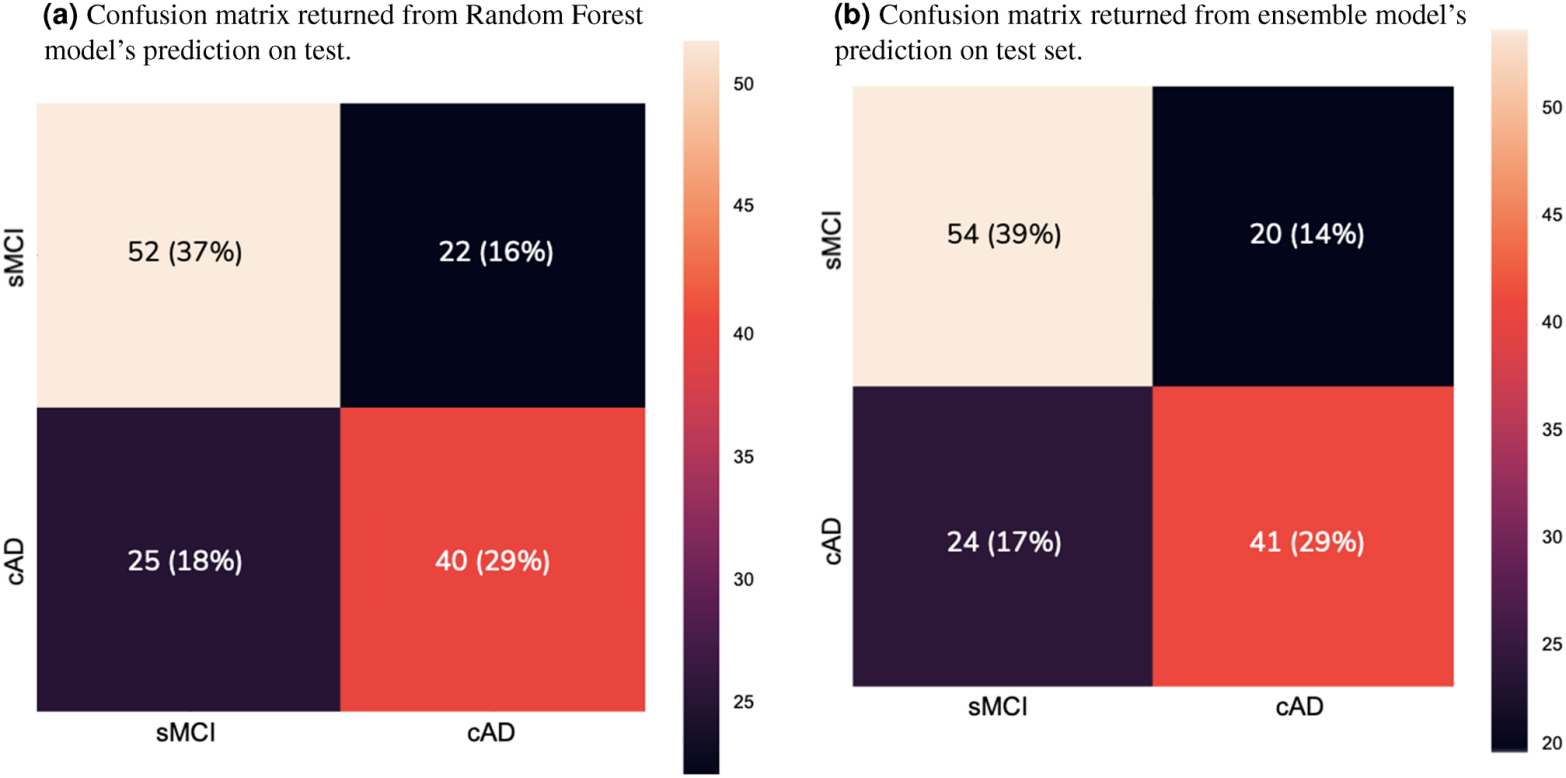
2 × 2 confusion matrices computed for the sMCI and cAD labels returned from prediction on test set compared with the co-occurrences of the observed outcome. The black and purple cells represent misclassified subjects, while the beige and red cells represent correctly classified subjects. The number of occurrences in each cell is given as number of subjects and percentage of the total test set for RF model and ensemble.

**Figure 2:**
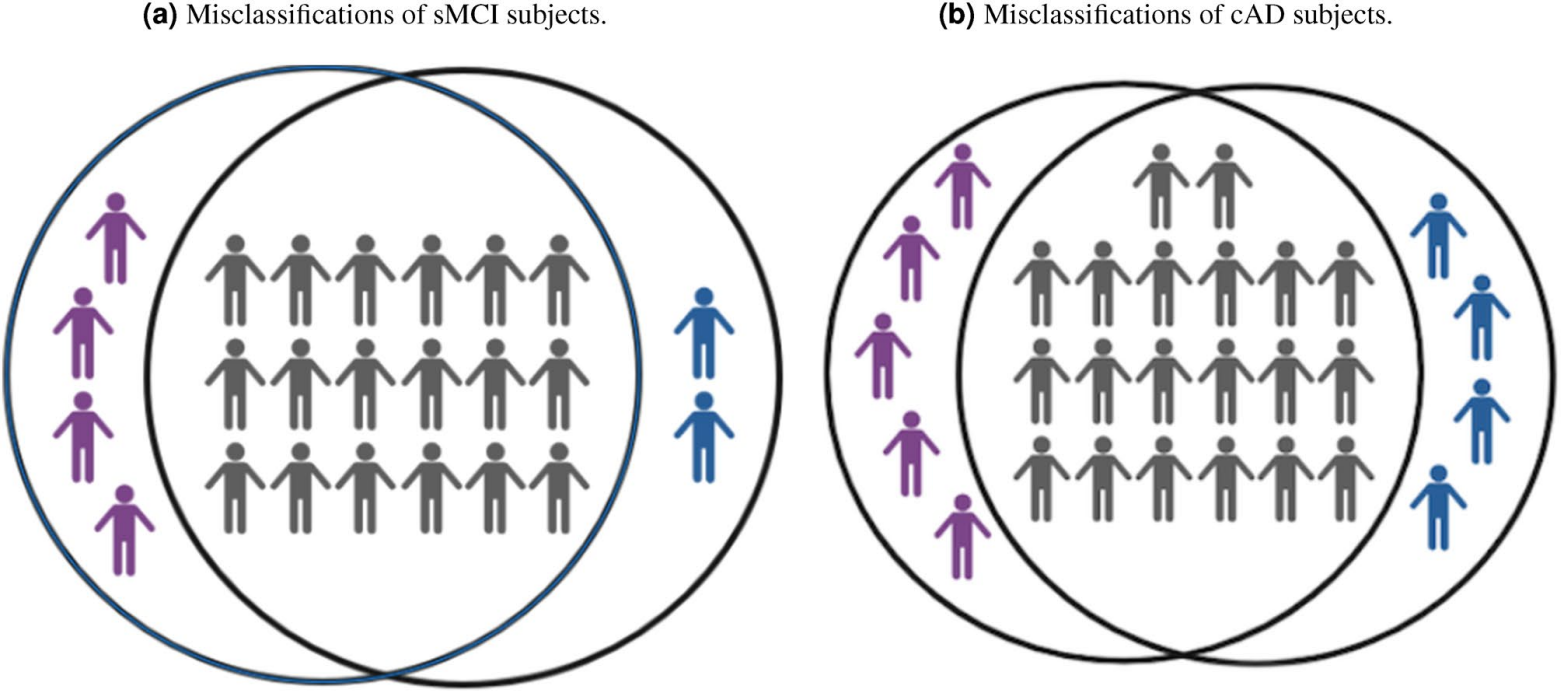
The figure illustrates the two models’ overlap in misclassified sMCI (**a**) og cAD (**b**). Gray symbols represent subjects for which the two models overlapped in misclassification. Purple and blue symbols represents additional subjects misclassified by the Random Forest model and the ensemble model, respectively.

Our top performing RF classification had an average accuracy of 74.6% (mean across 50 cross-validation folds). When the model was evaluated on our unseen test set, the overall classification accuracy was 66.2%. Similar results were obtained for the ensemble model. It achieved an average accuracy of 74.9% (mean across the same 50 folds), while evaluation on the test set yielded a somewhat higher accuracy of 68.3%. The 2 × 2 confusion matrices in Fig. 1 illustrate the correspondence between the true labels, the predictions returned from the RF model (Fig. 1a) and the predictions of the ensemble model (Fig. 1b). The RF model misclassified $\frac{22}{74}$ sMCI subjects as converters and $\frac{25}{65}$ cAD subjects as stable, resulting in a specificity of 70.3% and a sensitivity of 61.5%. The ensemble algorithm misclassified $\frac{20}{74}$ sMCI subjects as converters and $\frac{24}{65}$ cAD subjects as stable, resulting in a specificity of 73.0% and a sensitivity of 63.1%. As illustrated by Fig. 2, the two models largely overlapped in the subjects they misclassified: 18 of the same sMCI subjects (Fig. 2a) and 20 of the same cAD subjects (Fig. 2b).

Based on classification labels returned from the most accurate model (i.e. the ensemble model), comparative statistical analysis was conducted to investigate potential group differences between the four labels returned from the model. Table 2 shows that both groups of misclassified subjects (i.e. FP and FN) deviated from the correctly classified groups on several measures. Compared to correctly classified cAD, the group of misclassified cAD showed poorer results on all three memory tests and had smaller hippocampal volume. The same pattern, in addition to poorer score on TMTB, was evident for misclassified sMCI when compared to correctly classified sMCI, with FP showing cognitive impairments more similar to TP.

**Table 2:** Demographics, cognitive function and biological measures in correctly and misclassified patients.

	TN (n = 54)	FP (n = 20)	FN (n = 24)	TP (n = 41)	${p}<0.004^{A}$
	Mean (SD)	Mean (SD)	Mean (SD)	Mean (SD)	
**Demographics**					
Age	72.1 (7.32)	74.9 (7.19)	74.8 (8.01)	73.3 (7.61)	–
Gender (% F)	38.9	55.0	29.2	43.9	–
**Cognitive function**					
RAVLT-Im	39.15 (8.85)	30.60 (7.23)	35.38 (7.60)	28.12 (4.83)	a, b
RAVLT-delay	5.89 (3.41)	1.20 (1.54)	4.38 (3.00)	1.24 (1.55)	a, b
RAVLT-recog	12.15 (2.66)	9.40 (3.42)	11.71 (2.60)	8.66 (3.63)	a, b
TMTA	37.2 (13.1)	40.8 (8.3)	42.6 (28.9)	45.1 (25.8)	–
TMTB	91.6 (31.9)	129.6 (61.3)	130.0 (88.6)	134.4 (77.4)	a
CFT animals	18.69 (4.82)	16.80 (5.35)	16.00 (4.23)	15.81 (4.24)	–
GDS	1.82 (1.35)	1.85 (1.14)	1.29 (1.12)	1.51 (1.25)	–
ANART total errors	13.0 (9.7)	9.2 (7.3)	13.0 (9.9)	13.3 (10.3)	–
**Biological measures**					
Hippocampus volume	0.00457 (7.4*10$^{-4}$)	0.00384(6.1*10$^{-4}$)	0.00439 (6.2*10$^{-4}$)	0.00372 (6.8*10$^{-4}$)	a, b
APOE (% positive)	37.0	55.0	45.8	78.0	–

*TN* correctly classified sMCI, *FP* sMCI subjects misclassified converters, *FN* cAD subjects misclassified as stable, *TP* cAD subjects correctly classified, *RAVLT* Rey Auditory Verbal Learning Test, *TMT* Trail Making Test, *CTF* Category Fluency Test, *GDS* Geriatric Depression Scale, *ANART* American National Reading Test. $^{A}$Multiple comparisons abbreviated as: a = TN differ from FP; b = FN differ from TP. Group mean differences at Bonferroni corrected alpha level of 0.004 $(\alpha _{altered} =0.05/12 = 0.004$, rounded) considered statistically significant.

#### Importance of features

**Figure 3:**
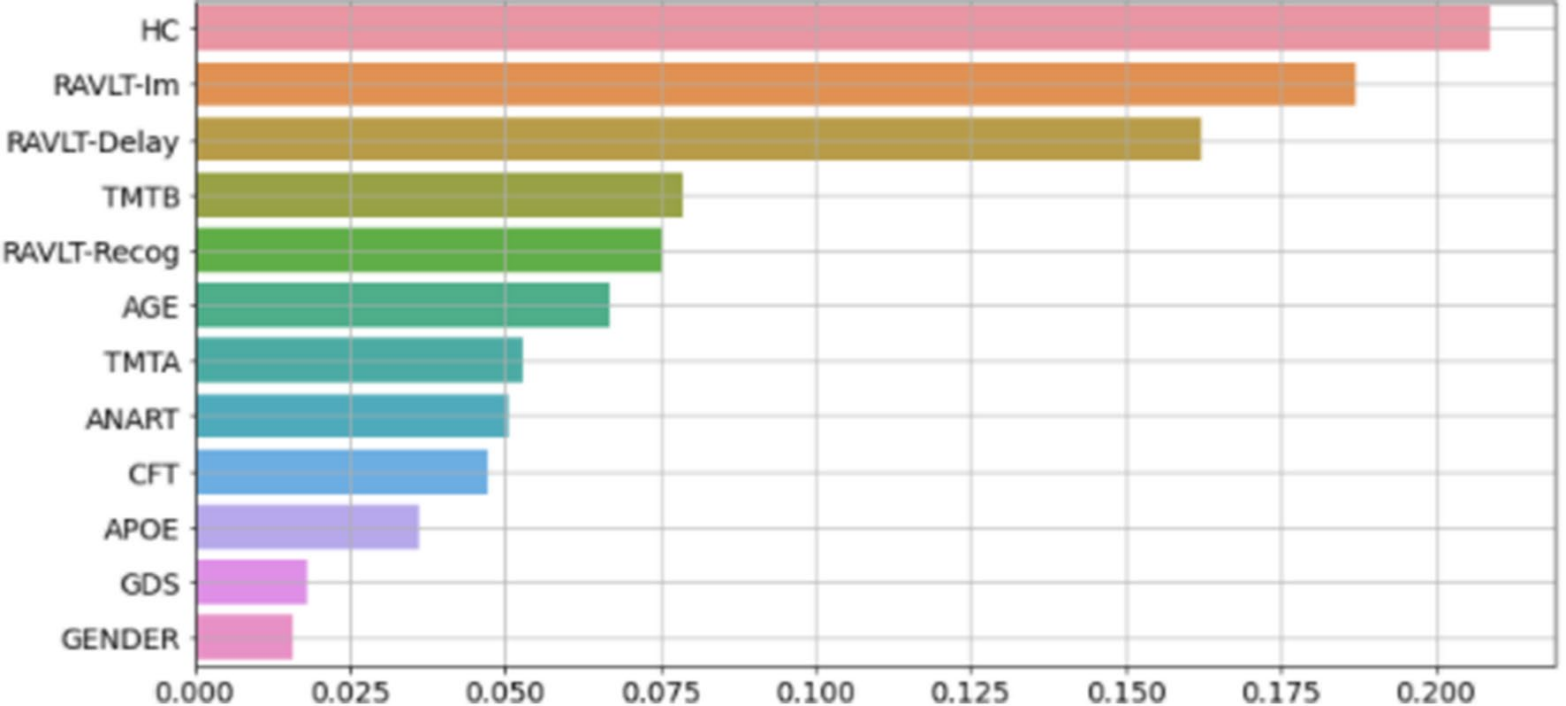
Feature importances calculated by decrease in impurity from evaluation on test set. All the predictors included in the model are displayed on the y-axis while the x-axis depicts their relative importance.

We used two different methods to investigate feature importance in the RF model. As illustrated in Fig. 3, the calculation of importance based on Mean Decrease in Impurity ranked hippocampus volume, RAVLT immediate and RAVLT delayed to be most important for prediction.

A model agnostic permutation importance test was also conducted. This algorithm shuffles each feature several times, with different permutations, while all the other features are kept constant. Table 3 shows the output of these calculations. Positive values means poorer predictions on shuffled data compared to real data, indicating that the feature contains information important for the prediction. Similarly to calculations of features importance based on decrease in Gini impurity, hippocampus volume and RAVLT immediate were ranked as the two most important features.

**Table 3:** The table depicts each feature’s importance in descending order calculated by permutation.

0.0403 ± 0.0503	HC
0.0245 ± 0.0413	RAVLT-Im
0.0086 ± 0.0108	AGE
0.0058 ± 0.0141	CFT
0.0000 ± 0.0129	GENDER
− 0.0014 ± 0.0211	APOE
− 0.0014 ± 0.0058	GDS
− 0.0029 ± 0.0503	RAVLT-delay
− 0.0058 ± 0.0168	TMTA
− 0.0101 ± 0.0147	TMTB
− 0.0129 ± 0.0058	ANART
− 0.0158 ± 0.0279	RAVLT-recog

The leftmost column in each row depict average effect on model accuracy by random shuffling ± how the accuracy varied from one reshuffling to the next. The two most important features are hippocampal volume and RAVLT immediate, followed by age, category fluency and gender.

## Discussion

AD, a progressive and multifactorial neurodegenerative disease, is identified by cognitive symptoms that tend to manifest itself long after the disease process is well established in the brain. Characterizing early markers of subsequent progression towards AD is therefore a paramount goal in the research field. The present study contributes to this endeavor by characterizing and separating two groups of patients identified with MCI at a baseline examination: one group that over time become AD patients (cAD) and one group remaining stable with an MCI diagnosis (sMCI). By running two independent classification methods within a machine learning framework, including information about cognitive function, total hippocampal volume and genetic APOE status from the baseline examination as features, we obtained a cross-validation accuracy of about 70% when classifying patients as belonging to one of the two predefined groups. This level of accuracy was confirmed across two different classification methods, which overlapped largely in their classifications at the subject-level. Impaired memory function was found to be a core symptom in MCI patients on a trajectory towards AD. Exploratory data analysis comparing results among correctly and misclassified individuals showed that sMCI patients who were falsely defined as AD converters tended to be more impaired than those who truly belonged to this group. A slow response-time and a high number (78%) of APOE-e4 carriers in the true positive group were also noted.

In line with previous studies characterizing the cognitive profiles of stable and converting MCI^27^, we found that the two groups could be differentiated already at the baseline examination, with the largest effects sizes associated with measures of episodic memory function and hippocampal volume. These measures were also given strong weights according to two different analytic approaches for feature importance. Although the RF generated feature importance and the permutation importance converged in the upper ranking, delayed recall was rated differently between the two estimates. This suggests that possible mutual information in memory tests should be controlled for when running such random shuffling^28^. Overall, these results confirm a close relation between memory function and hippocampus^29^, and that both tend to be affected already in an early stage of AD^4^.

Some interesting findings emerged from the exploratory analysis of correct and misclassified patients. Performance among the sMCI patients falsely classified as AD converters showed lower performance on tests of memory function as well as executive function (EF) than those correctly classified as sMCI. This may indicate that these patients would be diagnosed with AD in a longer time perspective. Together with the most severe impairment in the group correctly allocated to the cAD group, the results gave some support to include impairment of EF processes as prodromal symptoms of AD^30^, even when a patient originally was defined within the amnestic subgroup of MCI. It must, however, be underscored that a test of EF selected for the present study—the B version of the Trail Making Test—assesses only a fraction of the cognitive processes involved in EF^31^, and that other fractions may be more specific to other neurodegenerative diseases (see e.g.,^32^). The highest number of APOE positive subjects were recognised in the true and false positive subgroups, in line with conclusions from previous studies^9–11^. Despite these group level differences, APOE status did not have large discriminating power in the classification model. There are several plausible explanations for these findings. While the $\varepsilon 4$ allele is well-characterized as a risk factor in a dose-dependent manner, there are two other common alleles of the APOE gene; $\varepsilon 2$ and $\varepsilon 3$. The APOE $\varepsilon 3$ is considered neutral in terms of AD risk, but APOE $\varepsilon 2$ has been shown to have a protective effect^33^. Our operationalization of APOE genotype as binary a feature, i.e., negative (no $\varepsilon 4$ alleles) and positive (at least one $\varepsilon 4$ allele), may thus have resulted in the loss of important information, as subjects who were heterozygote with $\varepsilon 2$/$\varepsilon 4$ (neutral APOE profile) in the current study were grouped with subjects homozygote for $\varepsilon 4$ (highest risk APOE profile).

Taken together, our results confirmed that early prediction of AD is indeed a challenging task. Developmental trajectory in an individual patient is determined by numerous biological, lifestyle and environmental factors, and their interplay may all act as mediators of susceptibility for AD. This explains the large heterogeneity in both the pathological and clinical manifestations characterizing AD and other neurodegenerative diseases. Furthermore, pure AD pathology is the exception rather than the rule. Post-mortem examinations have shown that pure AD pathology is identified in only 3-30% (age-dependent) of patients with a clinical AD diagnosis^34^, and that high loads of AD pathology is found even in individuals without clinical symptoms of the disease^35–37^. Although the present study was not designed to investigate such heterogeneity, we find it intriguing that a high number of subjects were correctly predicted from two independent statistical models primarily including cognitive measures as predictors. The results should thus inspire further longitudinal studies to investigate cognitive as well as other mediators of trajectories from non-pathological to pathological aging^38,39^.

The size of the MCI group, the longitudinal design and the consistency of findings across analytic approaches are considered to be main strengths of the present study. We showed that two independent classification algorithms yielded comparable predictive accuracy as well as a large subject-specific overlap regarding patients who were misclassified. The re-analysis of the T1-weighted MRI images by the FreeSurfer longitudinal stream should be considered as another strength by increasing the reliability of the extracted hippocampus volume^40^. We will also underscore the importance of our careful selection of patients to be included in our study. While most studies using ADNI data restrict inclusion to one or two study phases or the reprocessed data made available by the ADNI project (and shared in e.g. the ’ADNIMERGE’ file), the present study included subjects across all four study phases. This gave us a relatively large longitudinal sample where we could select variables that are commonly included as part of a clinical examination of patients suspect of a neurodegenerative disease. There are several limitations that should be noted. Although we obtained a classification accuracy above chance level, it should still be described as modest. This is partly related to restrictions associated with the AD and MCI definitions in the ADNI dataset. As already mentioned, an AD diagnosis is defined “probable” until post-mortem examinations. It is therefore possible that some of the subjects defined as AD, and thus used as “ground truth” in this study, were misdiagnosed. The patients defined with an MCI diagnosis is also a heterogeneous group, clearly illustrated in the work of Edmond et al.^41^. Among patients defined by ADNI as aMCI, they described several subgroups. One of those did even show performance on cognitive tests and brain measures (MRI) within the limits of normal function. A range of other factors should therefore be included to potentially raise the sensitivity and specificity to a clinically acceptable level. It should also be underscored that the ADNI dataset mainly includes highly educated and motivated volunteers geographically restricted to North America. Finally, some limitations related to the analytic approach should be mentioned. Although we ensured complete independence between features used to define outcome and features used to train the classification model, we are aware of the circularity associated with conducting group analysis on the prediction labels for true and false classifications returned from the ensemble models. Hence, these results should be viewed as exploratory. It is also a limitation that information about participation length was not controlled for in the statistical models.

In conclusion, the present study showed challenges related to early identification of patients at risk of a trajectory from MCI to AD. Although existing treatment is still far from reversing already established pathological changes, it may slow down the disease progression (see e.g.,^5,42^). Thus, early identification is essential to treatment of a neurodegenerative disorder. The present study proposed a multi-modal machine learning framework that uses clinically relevant data to classify MCI subjects into a group remaining stable and a group progressing to AD. The complexity related to diagnosing AD is illustrated by studies investigating family physicians’ accuracy of dementia diagnoses, which shows sensitivity for detecting mild dementia to be especially poor (14–33%)^43^. Although the accuracy achieved in the current study is below what should be considered sufficient to enable direct implementation in clinical practice, we believe the study should inspire further work towards developing automated prognostic tools, with an ultimate aim to design a supportive aid for clinicians responsible for giving information about prognostics to individual patients.

## Methods

### Sample

Data were obtained from the Alzheimer’s Disease Neuroimaging Initiative (ADNI) database (adni.loni.usc.edu). The ADNI project was launched in 2003 as a public–private partnership, led by Principal Investigator Michael W. Weiner, MD. The primary goal of ADNI has been to investigate whether serial magnetic resonance imaging (MRI), positron emission tomography (PET), other biological markers, and clinical and neuropsychological assessment can be combined to measure the progression of mild cognitive impairment (MCI) and early Alzheimer’s disease (AD). ADNI consists of four study phases, and for the present study we included subjects across all these phases who according to ADNI’s criteria were defined as MCI at their baseline (first) assessment. The ADNI study was approved by the Institutional Review Boards at each participating ADNI site (see full list here: http://adni.loni.usc.edu). All procedures were performed in accordance with relevant guidelines and regulations, and informed consent was obtained from all subjects prior to enrollment. The current study was approved by the ADNI Data and Publications Committee (ADNI DPC). Data used in the present study were downloaded on November 9th 2020, and inclusion is thus restricted to subjects whose data was uploaded to the ADNI database prior to this date.

Using ADNI’s definition, a subject is diagnosed as MCI if the study participant (i) reports concern due to impaired memory function; (ii) obtains a Mini Mental State Examination (MMSE) score between 24 and 30; (iii) a Clinical Dementia Rating Scale (CDR) score of 0.5; (iv) a score lower than expected (adjusted for years of education) on the Wechsler Memory Scale Logical Memory II (WMS-II); and (v) reports preserved function of daily living. From this group of MCI subject we restricted inclusion to subjects that had a minimum of three study visits (i.e., baseline visit and at least two additional visits) and had undergone a minimum of three MRI examinations. This resulted in inclusion of 708 subjects who were further divided into two diagnostic groups defined according to their longitudinal diagnostic status. One group was defined as stable MCI (sMCI; N = 357, 52.7%), meaning that they met the applied ADNI criteria for MCI on all study visits (n = 381, age range at baseline = 55–91). The other diagnostic group was defined as converters to AD (cAD; n = 321, 47.3%), and included subjects who were diagnosed with MCI at their first study visit, but met the criteria for probable AD on a subsequent assessment (n = 327, age range at baseline = 55–88). AD was defined according to following criteria; (i) MMSE score between 20-26 (inclusive), (ii) CDR score of 0.5 or 1.0, and (iii) meeting the National Institute of Neurological and Communication Disorders and Stroke/Alzheimer’s Disease and Related Disorders Association (NINCDS-ADRDA) criteria for probable AD (McKhann et al., 1984). To ensure uniform application of diagnostic criteria across the more than 59 different study cites involved in ADNI, a Central Review Committee verified each individual subject’s conversion to AD.

### Feature selection

The rationale and motivation behind the selection of features in the present study was our aim of keeping the features clinically relevant and close to being a proxy of an initial clinical assessment of a patient presenting problems suspect of an MCI diagnosis.

#### Demographic characteristics

Gender and age at baseline assessment were included as demographic features.

### Neurocognitive features

#### Rey auditory verbal learning test (RAVLT)

RAVLT^44^ is a list learning test included to measure different aspects of verbal learning and memory function. In the first learning trial, a list of 15 nouns is read aloud by the test administrator at a rate of one word per second. Immediately after the first presentation, the subject is asked to freely recall as many of these 15 words as possible. This procedure, with reading and recall of the same list, is repeated for 4 more trials. A total score for immediate recall [‘RAVLT-Im’] is calculated by adding the number of words correctly recalled across all five trials. After a 30-minutes delay period filled with testing unrelated to the verbal content of RAVLT, the subject is again asked to recall the 15 words from the original list, and the number of correct responses is used as a measure of delayed recall [‘RAVLT-Delay’]. Immediately following this, a list including the 15 target words from the learning trials intermixed with 15 distractor words is presented to the subject who is asked to identify the target words. From this, a recognition [‘RAVLT-Recog’] score is derived from the sum of correct responses.

#### Trail making test (TMT)

TMT^45^ has two parts, where the first (TMT-A) is used as a measure of processing speed, and the second (TMT-B) as a measure of the cognitive flexibility aspect of executive function. In part A, a sheet of paper with printed numbers from 1 to 25 is presented to the subject. The subject is then instructed to use a pen to connect the numbers in ascending order, and encouraged to work as fast as they can. Part B is similar, but here the numbers (1–13) are intermixed with letters (A–L). The subject is instructed to connect these by switching between the ascending numerical and alphabetical order, putting a stronger load on cognitive flexibility than the TMT-A part. The total number of seconds used to complete part A [‘TMTA’] and part B [‘TMTB’] are used as measures of processing speed and executive function, respectively. Maximum scores are 150 and 300 for part A and B, respectively, as the subject was stopped if these time limits were exceeded.

#### Category fluency test (CFT)

CFT^46^ is used as a verbal test of executive function in the present study. In CFT, the subject is asked to generate as many words as possible belonging to a given semantic category (animals) within a time limit of 1 minute. In addition to assessing verbal ability, and more specifically lexical access ability^47^, the task require aspects of executive function^48^: the subjects must focus on the task at hand, select words meeting the condition of belonging to the semantic category, as well as inhibit repetitive responses.

#### Geriatric depression scale (GDS), short form

The short form of the GDS^49^ is a self-reported questionnaire designed to identify symptoms of depression, specifically in an elderly population. As participants obtaining a total GDS score [‘GDS’] between 6 and 15 were excluded from the ADNI sample, the total GDS scores in our selected sample range between 0-5. The score in individual participants is still used to assess severity of depression, because even symptoms below diagnostic threshold may affect cognitive function in older adults^50^. The form includes 15 items to which the subjects answer by circling “yes” or “no” based on how they felt the past week. Ten questions are positively oriented for depression (e.g., “Do you feel that your life is empty?”) and the remaining five questions are negatively oriented (e.g., “Are you basically satisfied with your life?”). All questions are weighted equally, with one point given for each answer indicative of depression (maximum 15 points).

#### American national adult reading test (ANART)

ANART^51^ is designed to obtain an estimate of premorbid intellectual function. Subjects are asked to read a list of 50 words that are printed on a sheet of paper. All words are irregular in that they do not follow phonological and orthographical rules, and they are graded in terms of difficulty of correct pronunciation. Because of this irregularity, correct pronunciation depends on previous familiarity with the words. Performance is assessed according to phonetic accuracy in pronunciation of each word. In the present study we used the total number of errors [‘ANART’] as a proxy for premorbid intellectual function, obtaining a baseline measure that is expected to be relatively preserved in the MCI patients included in the present study.

### MRI acquisition and brain segmentation

Acquisition of 1.5 T MRI (for ADNI 1) and 3.0 T MRI (for ADNI GO/2/3) data at each of the multiple ADNI sites followed a described standardized protocol developed by ADNI. See http://adni.loni.usc.edu/methods/mri-analysis/mri-acquisition for sequence details.

The MRI images from ADNI were originally processed with two different versions of FreeSurfer (v.4.3 and v.4.1) and made available through the ADNI database. In previous work we have shown that the use of different versions of FresSurfer may lead to a relatively large discrepancy in the atrophy estimations^23^. We therefore re-processed all the included MRI images using the same version of FreeSurfer (v.7.1.1), using the longitudinal stream of FreeSurfer^52^. In the longitudinal stream, an unbiased within-subject template space and image^53^ is created using robust, inverse consistent registration^54^. Several processing steps, such as skull stripping, Talairach transforms, atlas registration as well as spherical surface maps and parcellations are then initialized with common information from the within-subject template, significantly increasing reliability and statistical power^52^.

A measure of the total hippocampus volume [‘HC’] was derived by combining the volume of the left and right hippocampi. To reduce the effect of individual and gender differences in brain sizes, the volumes were normalized using the total intracranial volume measure (eTIV) estimated by FreeSurfer.

### APOE status

Blood samples were collected at baseline for APOE genotyping. As part of the ADNI study, samples were transported from each study site by overnight transport to the University of Pennsylvania Alzheimer’s Disease Biomarker Laboratory where the genotyping was carried out. In the present study, APOE status was divided into a binary variable [‘APOE’] allocating subjects having no (APOE negative) and subjects having at least one $\varepsilon$4 allele (APOE positive) into separate groups.

### Analytic approach

A core objective of our study was to provide a broad phenotypic characterization of the two MCI subgroups (i.e. cAD and sMCI) at baseline, and compare the groups on these characteristics. The groups were therefore checked for similarities and differences with respect to all features used as input to the classification algorithms. Student’s t test for independent samples was used for continuous variables, and Pearson Chi-Square test for nominal variables. Statistical analysis of the twelve included variables were Bonferroni corrected for multiple comparisons, with an alpha level of 0.004 ($\alpha _{altered} =0.05/12 = 0.004$, rounded) considered to be statistically significant. If exploratory analyses indicated deviation from a normal distribution and/or heteroscedasticity, non-parametric Mann–Whitney U tests were conducted.

#### Classification methods

We constructed two different machine learning models: (1) an ensemble based model constructed by combining five different models, and (2) a Random Forest (RF) model^25^. The results from the two models were compared, and the RF model used to identify feature importance^26^.

The RF was constructed, trained and evaluated using Python and Scikit-learn (v. 0.19). For the ensemble based model, we trained 15 different supervised algorithms using PyCaret^55^, an open source machine learning library for Python. From these 15 models, we selected the top five performing models. All analysis were conducted on a single workstation running GNU/Linux Ubuntu 20.04.2 LTS. See the accompanying code repository for details about the training and evaluation of our models, https://github.com/ingryy/AD_converison.

#### Evaluating performance

To estimate a model’s generalization ability it is vitally important to use separate data sets for model construction and model evaluation. In our work the complete sample (n = 678) was split into a training set comprising 79% (n = 539) used for training the model, while a test set comprising 21% (n = 139) was held aside to be used for a final evaluation of how well the model performs on unseen data. The split into training and test sets was stratified with respect to age, gender and class membership.

#### Hyperparameter optimization and K-fold cross validation

To find hyperparameter settings for the machine learning models we used model-specific parameter grids and a randomized grid search across 50 cross-validation folds. The cross-validation folds were defined to preserve the ratio of the two classes in each fold.

#### Feature importance and model interpretation

After establishing how well the RF model can classify the two MCI subgroups we further assessed the predictive importance of the twelve individual features included in the model. Tree-based models, including RFs, has a built-in assessment of feature importance based on Mean Decrease in Impurity (MDI). Through this method, each feature’s relative importance is calculated by assessing to which degree the feature decreases impurity at a splitting node, with higher purity implying the features to have higher discriminatory power.

However, MDI can artificially inflate the importance of features if predictor variables vary in measurement scales and/or number of categories^56,57^. We therefore additionally measured feature importance using permutation feature importance, a technique introduced by Breiman^25,58^. This method quantifies each features importance by randomly reshuffling each predictor variable (one at the time), while assessing how this affects model performance. As the random permutation breaks the true relationship between a given feature and the outcome, model accuracy will decrease when a feature with true predictive power is permuted, whereas permuting a non-informative feature will likely render model performance unchanged, or even improved. Note that when permuting one feature at the time, the interactions or dependencies between features are not considered. As a result, if two features held mutual information important for prediction, permuting one of them will not necessarily negatively affect model performance as the information is preserved in the other.

## Data Availability

The data used in the current study are available from Alzheimer’s Disease Neuroimaging Initiative (ADNI) upon application. See http://adni.loni.usc.edu/data-samples/access-data/ for more information. For details about the experimental pipeline used in the current study see https://github.com/ingryy/AD_converison.
